# Exploring the Photophysical Properties of Some Dextran-Iron Oxide Nanoparticle Composites

**DOI:** 10.3390/molecules30112290

**Published:** 2025-05-23

**Authors:** Ion Lungu, Tamara Potlog, Anton Airinei, Radu Tigoianu, Carmen Gherasim

**Affiliations:** 1 Laboratory of Organic/Inorganic Materials for Optoelectronics, Moldova State University, 60 Al. Mateevici St., D-2009 Chisinau, Moldova; ionlungu.usm@gmail.com (I.L.); tpotlog@gmail.com (T.P.); 2 Laboratory of Physical Chemistry of Polymers, Petru Poni Institute of Macromolecular Chemistry, 41A Grigore Ghica Voda Alley, RO-700487 Iasi, Romania; tigoianu.radu@icmpp.ro (R.T.); gherasim.carmen@icmpp.ro (C.G.)

**Keywords:** FTIR, UV–Vis and transient absorption spectroscopy, fluorescence and phosphorescence spectra

## Abstract

In this study, we report the synthesis and characterization of Fe_3_O_4_ nanoparticles coated with dextran. The structural and optical properties of the Dx:Fe_3_O_4_ synthesized composites were investigated by Fourier Transform infrared (FTIR) spectroscopy, X-ray diffraction (XRD) and UV–Vis absorption spectroscopy. For the first time in this paper, the photophysics of Dx:Fe_3_O_4_ composites in water is studied using fluorescence and phosphorescence molecular spectrometry. An analysis of the absorption spectra of the Dx:Fe_3_O_4_ composite reveals the broad absorption bands with maxima at wavelengths of 227 nm, 264 nm, and 340 nm. Dx:Fe_3_O_4_ composite nanoparticles in water exhibit strong fluorescence with a quantum yield of 0.24% in contrast to 0.07% for dextran. Phosphorescence spectra confirm the formation of new emission bands within the Dx:Fe_3_O_4_ solution evidenced by the maxima shift for both dextran and Dx:Fe_3_O_4_ composites.

## 1. Introduction

The field of organic–inorganic hybrid materials has experienced a substantial increase over the past two decades, and now stands among the most dynamic and promising areas in materials science [1,2,3]. The concept of combining organic and inorganic components into a single system, while seemingly straightforward today, presents substantial challenges due to the intrinsic differences in the chemical nature and compatibility between the two classes of materials. Overcoming these drawbacks determined the development of a wide range of hybrid architectures—from molecular assemblies to nanocomposites—that exhibit novel functionalities which are inaccessible to purely organic or inorganic systems. This hybrid approach opens new pathways for materials with synergistic properties, where organic components offer flexibility, processability, and functional diversity, while inorganic counterparts provide stability, magnetic or catalytic activity, and enhanced optical properties.

In particular, the investigation of the photophysical processes in nanostructured hybrid systems has emerged as a field of growing interest, especially for applications in optoelectronics [4], imaging [5], and biosensing [6]. In such systems, the organic moiety frequently governs solubility, biocompatibility, and surface interactions, while the inorganic core contributes to the magnetic, electronic, or photonic functionalities. Among inorganic nanomaterials, Fe_3_O_4_ (magnetite) has drawn attention due to its favorable properties, including biocompatibility, colloidal stability, low toxicity, and the ability to act as an electron donor via its Fe^2+^ state [7,8,9]. Its surface is rich in hydroxyl groups, enabling strong interactions with polymer coatings such as polysaccharides or synthetic copolymers [10,11,12,13].

Various natural and synthetic polymers, including chitosan [14] and poly(styrene-alt-maleic anhydride) [15], have been employed to stabilize iron oxide nanoparticles and improve their dispersion in aqueous media. These polymers contain functional groups—hydroxyl, amino, ether, or anhydride—that can adhere to the iron oxide surface through hydrogen bonding or coordination, forming a stable core–shell structure [16]. Such coatings not only reduce particle aggregation, but also offer routes for chemical modification and potential applications in biological recognition and drug delivery [17,18,19]. Notably, poly(styrene-alt-maleic anhydride) exhibits low toxicity, biodegradability, and functional versatility, making it a valuable candidate for biomedical applications, including targeting of pathogens such as Mycobacterium tuberculosis [20].

Among polymer-coated iron oxide systems, dextran-coated Fe_3_O_4_ nanoparticles (Dx:Fe_3_O_4_) represent a particularly promising hybrid material [21]. Dextran is a natural, hydrophilic polysaccharide widely used for its biocompatibility and colloidal stabilization capabilities [22,23]. While its chemical and biological roles are well documented, the influence of the dextran on the photophysical properties of Fe_3_O_4_ nanoparticles remains insufficiently explored [24,25]. Recent findings suggest that organic–inorganic interfaces may introduce enhanced optical properties through modulation of energy transfer, excited-state lifetimes, and emission pathways [26,27]. In such systems, the presence of a magnetic core may alter radiative and non-radiative decay channels, affecting the efficiency and dynamics of fluorescence and phosphorescence processes.

In this study, we aim to elucidate the photophysical behavior of dextran (Dx) and Fe_3_O_4_:Dx nanoparticles through a comparative spectroscopic study. We investigate their steady-state fluorescence and phosphorescence emission, analyze spectral shifts and band deconvolutions, and determine their emission lifetimes. The results highlight distinct modulations in emission intensity, spectral maxima, and lifetime components induced by the magnetic core, revealing complex energy dynamics at the organic–inorganic interface.

## 2. Results and Discussion

### 2.1. Structural and Chemical Composition of the Dx:Fe_3_O_4_ Composite

**Figure 1:**
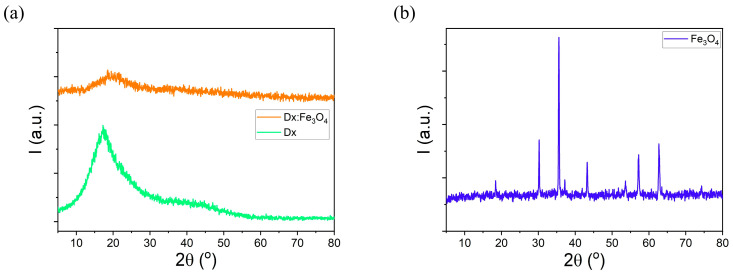
XRD patterns of (**a**) dextran, Dx:Fe_3_O_4_ composite and (**b**) Fe_3_O_4_.

Figure 1a shows XRD patterns for dextran and dextran-coated Fe_3_O_4_ with weight percentages of 1:1, while diffractogram of pure Fe_3_O_4_ NPs is presented in Figure 1b. The diffraction peaks of pure Fe_3_O_4_ NPs are observed at 2θ = 18.42°, 30.24°, 35.58°, 43.23°, 53.61°, 57.17°, and 62.75°, corresponding to the following diffraction planes: (111), (220), (311), (400), (422), (511), and (440), respectively. These diffraction peaks are in good agreement with the cubic spinel structure of Fe_3_O_4_ nanoparticles (JCPDS card No. 19-629) [23,28,29]. Sharp diffraction peaks suggest that the Fe_3_O_4_ nanoparticles are high crystalline. No peak related to any impurity was observed. In a dextran-coated Fe_3_O_4_ NPs diffractogram, a broadening of the diffraction peaks was noticed. As shown in Figure 1a, the intensities of the diffraction peaks decreased with the addition of Fe_3_O_4_ and their widening increased. It can be seen from the XRD pattern of the dextran-coated Fe_3_O_4_ that the position of the diffraction peaks does not change. The crystal phase of the Fe_3_O_4_ nanoparticles does not change after coating with dextran, they are only slightly shifted to higher 2θ degrees. The shift to higher diffraction angles suggests a decrease in the interplanar spacing, taking into account the interaction of the dextran chains with Fe_3_O_4_ nanoparticles. The XRD pattern of the dextran-coated Fe_3_O_4_ NPs demonstrates that the crystal structure of Fe_3_O_4_ does not change after surface modification, but confirms the interaction between dextran and Fe_3_O_4_ and their complex formation.

The average crystallite size (D) of Fe_3_O_4_ nanoparticles was estimated using the Scherrer relation: D = kλ/β cosθ (where λ is the X-ray wavelength (1.5408 Å), β represents the full width at half-maximum of the diffraction peak (FWHM), θ is the diffraction angle and k is Scherrer constant) [30]. The crystallite size obtained based on this relation was found to be about 41.1 nm for Fe_3_O_4_ nanoparticles. The diffraction pattern of dextran exhibits a broad diffraction maximum located at 17.19°. The average crystallite size of dextran: Fe_3_O_4_ NPs was around 10 nm. We can conclude that the coating process has a significant role in decreasing the crystallite size of the nanoparticles. The characteristic peak at 19.67° in the diffractogram of the dextran:Fe_3_O_4_ composite suggests that the dextran was incorporated on the surface of the Fe_3_O_4_ nanoparticles. Also, the decrease in intensity of the diffraction peaks in dextran:Fe_3_O_4_ is due to the in situ addition of the dextran on the surface of the magnetite nanoparticles leading significantly to the decrease in the crystallite size. The analysis of the XRD patterns reveals the amorphous/crystalline nature of the dextran coated iron oxide nanoparticles.

**Figure 2:**
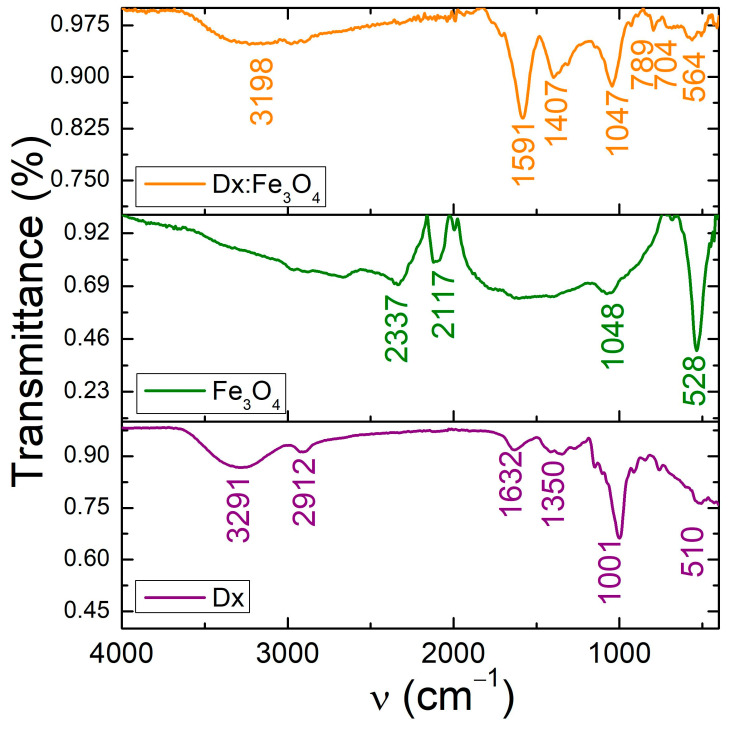
FTIR spectra of dextran, Fe_3_O_4_ and Dx:Fe_3_O_4_ composite.

To confirm the formation of the dextran-modified Fe_3_O_4_, also the FTIR spectra of pristine dextran, Fe_3_O_4_ NPs and dextran coated Fe_3_O_4_ nanoparticles were studied. Figure 2 shows the FTIR spectra of all samples for comparison. The two lower intense peaks observed between 564 cm^−1^ and 630 cm^−1^ are attributed to the stretching vibration mode associated with the metal–oxygen Fe–O bonds in the crystalline lattice of Fe_3_O_4_. Also, FT-IR spectrum of Fe_3_O_4_ NPs exhibits the broad absorption peak at about 3400 cm^−1^ that can be related to the presence of hydroxyl groups. It is noted that most of the absorption bands of dextran-coated Fe_3_O_4_ nanoparticles correspond to those of Fe_3_O_4_ and dextran. The characteristic absorption band of the Fe-O bond was found around 528 cm^−1^ for pure Fe_3_O_4_ NPs, as it is the signature of the metal–oxygen bonds in tetrahedral sites of Fe_3_O_4_ [31,32,33]. The absorption band of dextran around 510 cm^−1^ corresponds to the skeletal deformation vibrations of the glucose units from dextran. After the coating of the Fe_3_O_4_ NPs with dextran, the absorption band is shifted to 564 cm^−1^ due to the Fe-O stretching vibration, suggesting coordination between the iron ions and hydroxyl groups of dextran. The absorption bands of dextran at about 1350 cm^−1^ and 1632 cm^−1^ appear from –CH_2_ deformation vibration and H-O-H bending vibration [34,35]. In the dextran coated Fe_3_O_4_ NPs, the absorption band of dextran at 1632 cm^−1^ is shifted to 1591 cm^−1^ lower wavenumbers, indicating the changes in environment due to direct interactions between iron oxide and dextran backbone [8,28]. Comparing spectra of Fe_3_O_4_ and Dx: Fe_3_O_4_ NPs, some new absorption bands have appeared. For instance, the band at about 1591 cm^−1^ was due to the stretching vibration of the alcoholic hydroxyl (C–O), and the band at 1407 cm^−1^ was attributed to the bending vibration of C–H bond. These data proved that the surface of Fe_3_O_4_ NPs has been covered with dextran polymer. It is believed that different interactions such as van der Waals force, hydrogen bonds, and electrostatic interactions keep dextran on the surface of Fe_3_O_4_ NPs. As shown in Figure 2, the FTIR spectrum of dextran exhibit the broad absorption band at 3291 cm^−1^ indicating the presence of phenolic O–H group, while in Dx: Fe_3_O_4_ NPs, this band is shifted to 3198 cm^−1^. This reveals that dextran functional hydroxyl (–OH) groups offer an easy point for chemical conjugation with the Fe atoms on the magnetite surface, and render a partial single bond character to the C=O bond, weakening it, and shifting the stretching frequency to a lower value.

The changes in O-H and Fe-O absorption bands suggest that the dextran coating influences the hydration state and chemical environment of the magnetic core, providing new functional properties to the composite material. The attachment of dextran on Fe_3_O_4_ NPs surface was confirmed by Fourier transform infrared spectroscopy.

So, we propose that dextran-coated Fe_3_O_4_ NPs are produced by the reaction between the iron atom of Fe_3_O_4_ and oxygen in dextran. This polar covalent bond can be argued by the electronegativity difference of 1.8 for iron and 3.5 for oxygen. Because the difference between electronegativity has the value of 1.7, smaller than 2, the results show that the bond in dextran-coated Fe_3_O_4_ NPs is polar covalent. The 541 cm^−1^ peak characteristic of the Fe-O bond confirms the formation of the iron oxide nanoparticles, while 1407 cm^−1^ and 1591 cm^−1^ absorption bands assigned to the dextran δ(C–H) and ν(C–H) vibrational modes demonstrate dextran coated-Fe_3_O_4_ nanoparticles.

### 2.2. Electronic Transitions in Dx:Fe_3_O_4_ Composite

**Figure 3:**
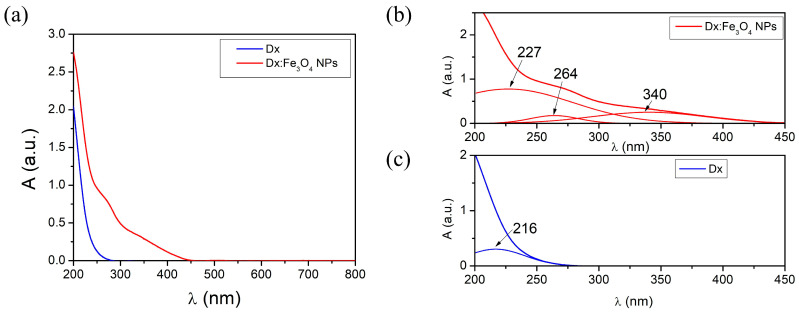
UV–Vis absorption spectra of (**a**,**c**) Dx and (**b**) Dx:Fe_3_O_4_ composite.

The UV–Vis absorption spectra exhibit significant findings for both dextran and Dx:Fe_3_O_4_ systems (Figure 3). Both samples exhibit strong absorption in the UV (200–350 nm) range of the electromagnetic spectrum, which is characteristic of high-energy electronic transitions, particularly π-π* transitions associated with the hydroxyl groups and glycosidic bonds present in dextran. In addition, as can be seen from Figure 3a, the absorption profile at 270–350 nm shows the presence of the plasmon band of iron oxide nanoparticles [33]. The increase in absorbance for the Dx:Fe_3_O_4_ system as compared to pristine dextran indicates that the coating enhances the light-absorbing characteristics of the composite [36,37]. The deconvolution of the absorption spectrum of dextran (Figure 3a) shows an absorption band at 216 nm, which aligns with typical absorption features of polysaccharides in this region. This band is attributed to the electronic transitions of the functional groups in the dextran structure, highlighting its interactions with the solvent and providing insights into its molecular environment.

The absorption spectrum of the Dx:Fe_3_O_4_ composite shows absorption maxima at 227 nm, 264 nm, and 340 nm (Figure 3b). The absorption band observed in the Dx:Fe_3_O_4_-NPs at 227 nm is close to the value of the absorption band in pure dextran (216 nm). This shift to higher wavelengths can occur due to interactions between the dextran molecules and the Fe_3_O_4_ nanoparticle surface, which modify the electronic environment of the polymer responsible for the absorption. Therefore, this absorption band reflects not only the presence of dextran, but its interaction with the Fe_3_O_4_ surface, potentially stabilizing or modifying its electronic states and confirming the successful coating of Fe_3_O_4_ with dextran, providing insight into how the coating affects the optical behavior of the system. The absorption bands of the Dx:Fe_3_O_4_ composite highlighted at 264 nm and at 340 nm arise from surface plasmon resonance phenomena and charge transfer transitions [38]. The Fe^2+^ and Fe^3+^ ions in the Dx/Fe^2+^Fe^3+^ precursor react with the hydroxyl groups (–OH) in the basic media, in which the Fe_3_O_4_ nanoparticles formed in the crosslinked dextran matrix, leading to the formation Dx:Fe_3_O_4_ composite.

### 2.3. Photophysical Properties in Dx:Fe_3_O_4_ Composite

**Figure 4:**
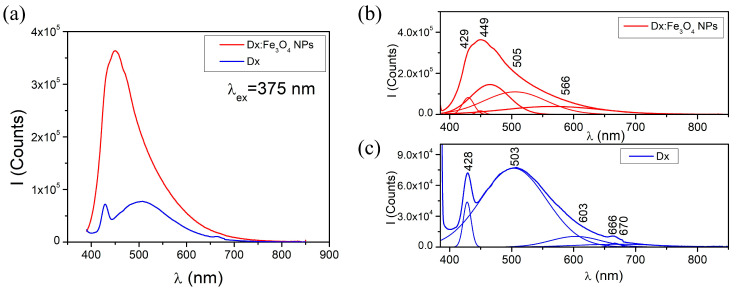
Fluorescence spectra of (**a**,**c**) Dx and (**b**) Dx:Fe_3_O_4_ composite (λ_ex_ = 375 nm).

The fluorescence spectra were recorded both for the pure dextran and Dx:Fe_3_O_4_ composite, under 375 nm excitation (Figure 4). The fluorescence emission bands for dextran are seen at 428 nm and 524 nm when excited at 375 nm. The spectra reveal distinct differences between dextran and Dx:Fe_3_O_4_ composites, indicating the presence of new emission centers, altered energy levels due to the dextran coating on the Fe_3_O_4_ NPs, and an increase in fluorescence intensity. Fluorescence in these materials occurs from electronically excited states back to the ground singlet state, releasing photons in the process. The deconvolution of the spectrum, for pure Dx, presented in Figure 4c) shows emission maxima at 428, 503, 603, and 666 nm, indicating multiple emission centers likely arising from the structural features of the dextran. The 428 nm band can be attributed to π-π* electronic transitions, typical for molecules with conjugated systems, where electrons are excited to higher energy levels and return to the ground state through photon emission [39]. The emission band situated at 503 nm results from n-π* transitions, associated with non-bonding electron pairs (e.g., on oxygen in glycosidic bonds). These transitions generally require less energy than π-π* transitions, which explains the emission at a longer wavelength [40]. The maxima at 603 and 666 nm are attributed to low-energy vibrational transitions, suggesting that the complex polysaccharide structure of dextran contains vibrational frequencies contributing to these emissions [28].

For the Dx:Fe_3_O_4_ composite, the fluorescence spectrum exhibits notable changes, with new maxima appearing at 429 nm, 449 nm, 505 nm, and 566 nm (Figure 4b). The 429 nm maximum represents a slightly shifted π-π* transition like that of pure Dx, suggesting an electronic interaction between the nanoparticles and dextran’s functional groups, which slightly alters the transition energy. A new emission band at 449 nm represents modified n-π* transitions, potentially due to increased spin–orbit coupling introduced by Fe_3_O_4_, known as the heavy-atom effect. The 505 nm maximum suggests that the glycosidic bond characteristics of dextran are kept, but they are influenced by the presence of Fe_3_O_4_ NPs. Finally, the 566 nm maximum implies a charge-transfer transition between Fe_3_O_4_ and dextran, where electrons are transferred between the organic ligand (dextran) and the Fe_3_O_4_ NP, creating lower-energy transitions characteristic of ligand-to-metal charge transfer. Such phenomena are typical for systems with strong surface interactions between organic and inorganic components [38]. Overall, these changes in the fluorescence spectrum indicate that Fe_3_O_4_ nanoparticles influence the electronic states of dextran, creating new emission bands due to enhanced ISC and altered vibrational states.

**Figure 5:**
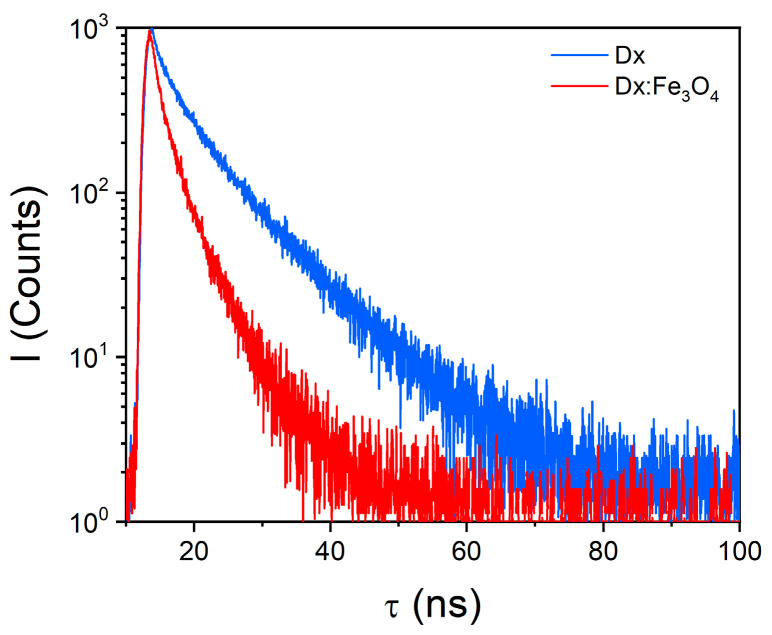
Fluorescence decays of dextran and Dx:Fe_3_O_4_ composite (λ_ex_ = 375 nm).

The fluorescence lifetime measurements were also performed (Figure 5). In Table 1, the values of fluorescence lifetimes and the quantum yield of fluorescence are shown. As one can see, both pure dextran and Dx:Fe_3_O_4_ composite display a bi-exponential decay model, with lifetime values of 2.18 ns and 9.24 ns, and a low quantum yield of 0.07% for dextran (Table 1). In contrast, the fluorescence decay detected in the Dx:Fe_3_O_4_ composite do not change significantly. The Fe_3_O_4_ nanoparticles coated with dextran exhibit lifetime values of 1.18 ns and 4.44 ns, respectively, with an enhanced quantum yield of 0.24%. Analyzing the fluorescence lifetimes of the dextran and Dx:Fe_3_O_4_ composite, it can be observed that the fast component of the lifetime has a lower amplitude (27.41%), whereas for Dx:Fe_3_O_4,_ the two contributions are practically equal (Table 1). The shorter fluorescence lifetimes observed in Dx:Fe_3_O_4_ suggest that the coating process influences the electronic environment, due to altered molecular interactions between Fe_3_O_4_ and dextran and the average time that the molecule spends in the excited state decreases.

The prolongation of a molecule’s fluorescence lifetime was attributed to the presence of relatively rigid excited-state structures with no rotatable bonds. By increasing or decreasing the electronic density of the substituents, the charge distribution and charge transfer processes within the Dx:Fe_3_O_4_ molecule can be controlled, leading to distinct excited states and nonradiative pathways.

The increase in the quantum yield of Dx:Fe_3_O_4_ as compared to pure dextran suggests more efficient fluorescence processes, possibly due to modified electronic states introduced by the Fe_3_O_4_ core. This enhancement in fluorescence yield, combined with distinct fluorescence lifetimes, confirms that dextran’s interaction with Fe_3_O_4_ alters its emission properties, making Fe_3_O_4_ a promising candidate for applications where controlled fluorescence behavior is desired.

**Figure 6:**
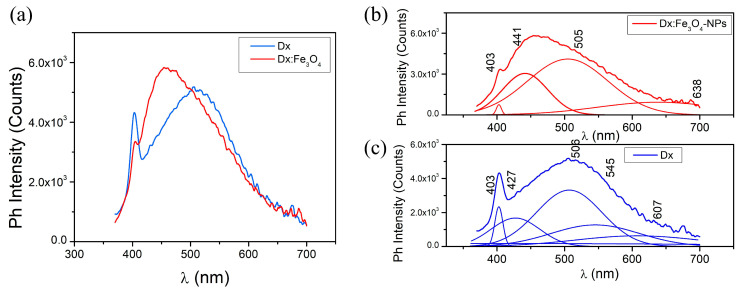
Phosphorescence spectra of (**a**,**c**) Dx and (**b**) Dx:Fe_3_O_4_ composite (λ_ex_ = 355 nm).

Phosphorescence measurements for both pure Dx and Dx:Fe_3_O_4_ are shown in Figure 6. Phosphorescence spectra for both Dx and Dx:Fe_3_O_4_ display similar forms of spectrum, with exception that maximum of Dx:Fe_3_O_4_ shift to shorter wavelengths. The deconvolution of the phosphorescence spectra for pure dextran reveals emission maxima at 403, 427, 506, 545, and 607 nm (Figure 6c). The emissions at longer wavelengths reflect vibrational relaxation within the triplet state, where the molecule’s complex structure allows the emission of lower-energy photons over extended times. The band at 427 nm corresponds to a direct transition from T_1_ to S_0_, while emissions at 506, 545, and 607 nm represent lower-energy relaxations that contribute to dextran’s broad phosphorescent signature. Such emission bands indicate multiple relaxation pathways in dextran, due to its complex polysaccharide structure, which enables a variety of vibrational and electronic transitions within the triplet state [41,42].

The deconvoluted spectrum of Dx:Fe_3_O_4_, shows that bands situated at 607 nm shift to 638 nm, red region. The red shift from 427 nm to 441 nm and the appearance of a band at 638 nm suggest that Fe_3_O_4_ stabilizes the triplet states, creating additional low-energy states for phosphorescent emission.

This behavior can be attributed to the enhancement of intersystem crossing (ISC), a spin-forbidden process that is typically inefficient in organic systems lacking heavy atoms or external perturbations [43]. In the Dx:Fe_3_O_4_ system, the Fe_3_O_4_ magnetic core enhances ISC through several mechanisms. First, the presence of Fe^2+^ and Fe^3+^ ions introduce localized *d* orbitals and unpaired electrons, which generate magnetic dipole interactions at the organic–inorganic interface. These interactions act similarly to spin–orbit coupling, facilitating otherwise forbidden singlet–triplet transitions. Second, the magnetic field generated by the Fe_3_O_4_ core can promote spin inversion and alter relaxation pathways, resulting in a higher population of the triplet states.

**Figure 7:**
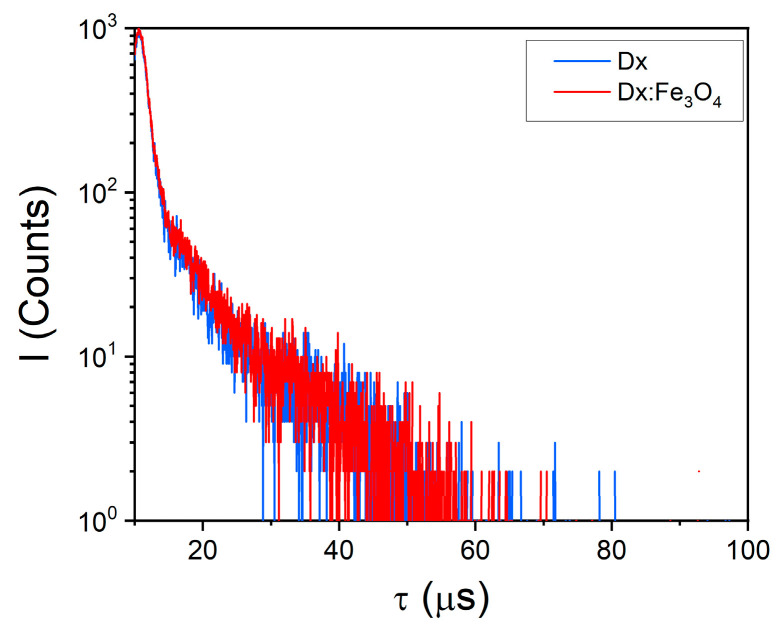
Phosphorescence lifetimes of dextran and Dx: Fe_3_O_4_ composite.

Phosphorescence lifetimes presented in Figure 7 further validate these observations. Pure dextran shows lifetimes of 1.09 μs and 8.83 μs, while Fe_3_O_4_ shows lifetimes of 1.10 μs and 8.46 μs. The slight reduction in lifetime for Dx:Fe_3_O_4_ suggests additional non-radiative decay pathways introduced by the Fe_3_O_4_ surface. These pathways may be due to Dx energy transfer, where Fe_3_O_4_ facilitates electron movement, shortening triplet-state lifetimes while maintaining phosphorescence emission. Together, the shifts in emission maxima and lifetime changes reveal that Fe_3_O_4_ enhances ISC and modifies energy decay, making Fe_3_O_4_ a promising material for applications requiring stable, prolonged emission.

**Figure 8:**
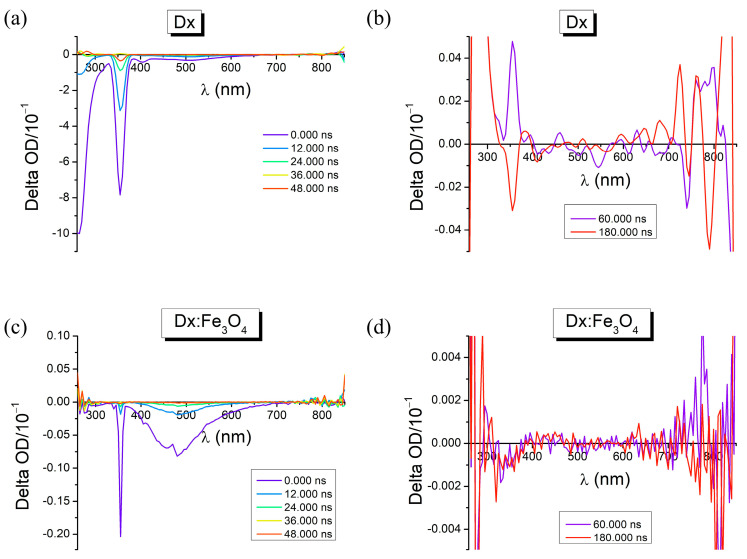
Transient absorption spectra of (**a**,**b**) Dx and (**c**,**d**) Dx:Fe_3_O_4_ composite.

In order to demonstrate the excited-state processes and the involvement of the higher energy electronic states (S_n_ > 1), transient absorption spectroscopy (TA) was used. Laser flash photolysis was performed using short laser pulses at 355 nm. The transient absorption (TA) spectra for the Dx and Dx:Fe_3_O_4_ composite are shown in Figure 8. In the transient absorption map at ns for Dx, it can be noticed ground state bleaching bands (GBS) at 260 and 280 nm, absorption in the excited state (ESA) at 255 and 275 nm, respectively, and more than one excited state (S_n_ > 1). At longer wavelengths, such as 405 and 505 nm, stimulated emission (SE) occurs. Also, for Dx:Fe_3_O_4_ composite, it can be noticed ground state bleaching bands (GBS) at 260 and 300 nm, absorption in the excited state (ESA) at 250 and 270 nm, and at longer wavelengths, such as 460 and 480 nm stimulated emission (SE) occurs.

The spectra contain a prominent peak with negative change in optical density at wavelengths corresponding to the excited energy of the laser as the delay between the pump and the probe pulse was increased from 0 ns to 48,000 ns. This bleach feature is attributed in dextran to the photoexcited species because of the degeneracy of the LUMO band and higher effective mass of the dextran. In addition, the spectra contain a much weaker photoinduced absorption-magnified in amplitude in Figure 8a,c which is attributed to trapped in dextran. Accordingly, this feature increases within 60,000 ns corresponding to trapping at surface of dextran. When dextran loaded to Fe_3_O_4_, the very weak negative feature between 400 nm and 600 nm observed in dextran increases and broadens to 700 nm. With increasing the delay between the pump and the probe pulse to 180,000 ns, the localized surface plasmon resonance takes part in both with high intensity in pure dextran.

**Figure 9:**
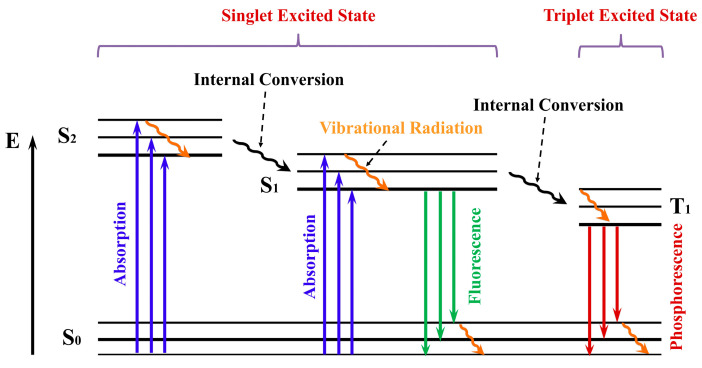
Diagram presenting the absorption and emission processes in Dx:Fe_3_O_4_ composite.

The Jablonski diagram for Dx:Fe_3_O_4_ illustrates the key electronic states and transitions that occur following light absorption (Figure 9).

Upon excitation, electrons from Fe_3_O_4_ are promoted from the ground singlet state (S_0_) to an excited singlet state (S_1_ or possibly higher states such as S_2_) as shown in Figure 9. Once in the excited state, electrons undergo rapid relaxation to the lowest singlet excited state (S_1_) via internal conversion (IC). From S_1_, they can return to the ground state (S_0_) by emitting light as fluorescence, a process typically characterized by nanosecond lifetimes. However, the presence of Fe_3_O_4_ in the composite nanoparticle significantly enhances intersystem crossing (ISC) from the singlet state (S_1_) to the triplet state (T_1_) due to the heavy-atom effect, where spin–orbit coupling introduced by Fe_3_O_4_ facilitates spin flip transitions. In the triplet state, electrons present longer lifetimes (microsecond), allowing for delayed photon emission through phosphorescence as they transition back to S_0_. In Dx:Fe_3_O_4_, the Fe_3_O_4_ the energy landscape modifies, resulting in red-shifted emissions and the appearance of new lower-energy states, evidenced by peaks at 441 nm and 538 nm in the phosphorescence spectrum. This Jablonski diagram thus reveals the unique role Fe_3_O_4_ plays in enhancing ISC and stabilizing triplet states, leading to modified fluorescence and phosphorescence behaviors in the Dx:Fe_3_O_4_ system.

## 3. Materials and Methods

### 3.1. Materials

Dextran (M_w_ = 40,000 Da), Fe_3_O_4_, hydrogen peroxide, acetic acid was procured from Merck, Germany, without further purification. In all experiments, double distilled water was utilized. The solvents were acquired from Sigma-Aldrich, St. Louis, MO, USA, and they were of spectrophotometric grade.

### 3.2. Synthesis

**Figure 10:**
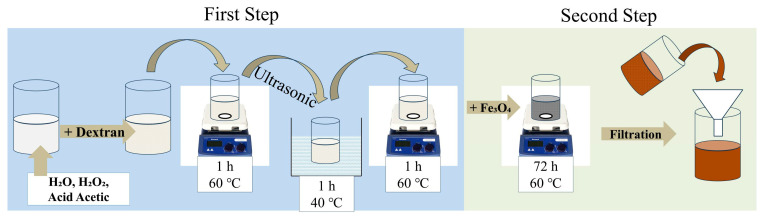
Schematic representation of coating of Fe_3_O_4_ NPs with dextran.

The synthesis of iron oxide nanoparticles was performed using the co-precipitation method described previously [23]. The preparation of the Dx:Fe_3_O_4_ composite was carried out through a two-step process (Figure 10). In the first step, dextran with a molecular weight of 40 kDa was degraded. For this, 2 g of dextran were dissolved in 196 mL of distilled water, 4 mL of acetic acid (99.9%), and 1 mL of hydrogen peroxide (H_2_O_2_) with a concentration of 60% to facilitate the degradation of dextran. H_2_O_2_ is a strong oxidizing agent that break down large polymers into smaller fragments through an oxidation process. The mixture was initially homogenized using a magnetic stirrer for one hour at a temperature of 60 °C. Subsequently, the reaction vessel was transferred to an ultrasonic bath, where the mixture was subjected to ultrasonication for 1 h to ensure the breakdown of the high-molecular-weight polymer, dextran, into low-molecular-weight fragments. After the ultrasonic treatment, the mixture was again homogenized using the magnetic stirrer for one hour, maintaining the temperature at 60 °C. Once the dextran degradation was completed, 1 g of Fe_3_O_4_ was added to the solution, and stirring was continued for 72 h at the same temperature of 60 °C. After this stirring period, the resulting solution was cooled to room temperature, filtered using filter paper to remove any impurities and undissolved particles. Finally, a dark brown solution was obtained, indicating the formation of Dx:Fe_3_O_4_ composite.

**Figure 11:**
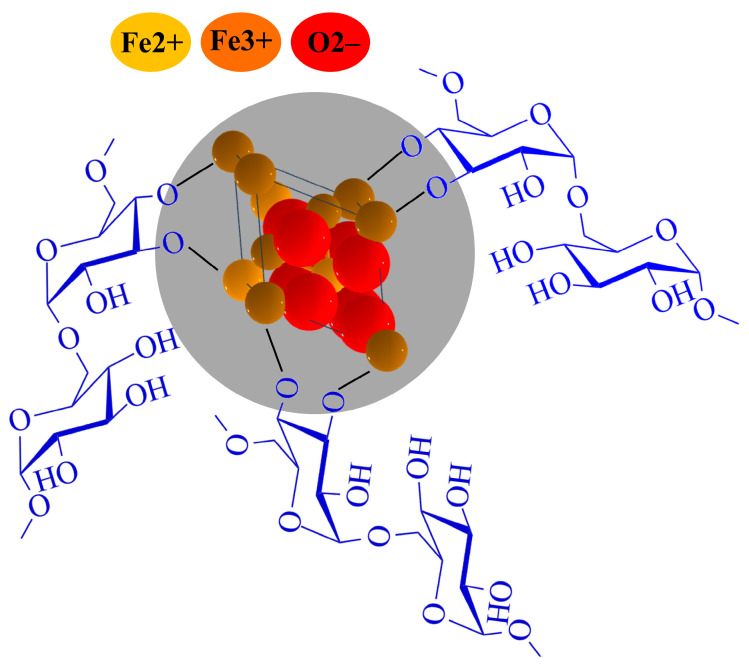
Molecular structure of Dx:Fe_3_O_4_ composite.

Due to the ferroelectric nature of Fe_3_O_4_, the particles tended to be attracted to the magnetic stirrer at low speeds; therefore, we increased the stirring speed to approximately 600 rpm. This high speed prevented the accumulation of particles around the stirrer and maintained a uniform suspension throughout the entire synthesis process. The attraction of free Fe_3_O_4_ NPs from the dextran solution by the magnetic stirrer indicates whether the Fe_3_O_4_ NPs were coated with dextran and where are not. Therefore, the stirrer was periodically turned off to check how much Fe_3_O_4_ interacted with dextran. This periodic monitoring was essential to ensure that the Fe_3_O_4_ NPs were effectively packed with dextran. Figure 11 illustrates the molecular structure of Dx:Fe_3_O_4_ composite.

### 3.3. Methods

The products were characterized using a FTIR spectrometer (Bruker ALPHA, Vienna, Austria) in the range of 350–4000 cm^−1^. The spectral data were processed using OPUS v. 7.5 or OMNIC v. 9.1 software. XRD analysis was carried out on a SHIMADZU XRD 6000 (Shimadzu, Tokyo, Japan) diffractometer using Ni-filtered CuK_α_ radiation (λ = 15,418 Å), with a step scan of 0.02°, a counting time of 1 s/step, scan step of 0.02°, angular range between 20 and 80°. The electronic absorption spectra were measured by an UV–Vis spectrophotometer (Lambda 25, Perkin Elmer, Shelton, CT, USA) using 10 mm quartz cuvettes. Fluorescence measurements were performed on LS55 luminescence spectrometer (Perkin Elmer, Shelton, CT, USA). Time-correlation single photon counting system (FLS 980, Edinburgh Instruments, Edinburgh, UK) was employed to conduct the time-resolved photoluminescence analysis using a nanosecond diode laser at 375 nm as excitation source. The absolute fluorescence quantum yield was estimated by FSL 980 integrated sphere using solutions having the absorbance below 0.1 and excitation wavelengths corresponding to the absorption band maximum. For the transient absorption spectra, the determinations were performed with LP980 laser flash photolysis spectrometer (Edinburgh Instruments, England), using a Nd YAG laser at excitation wavelength of 355 nm. Experiments were carried out at room temperature in 10 mm quartz cells.

## 4. Conclusions

Finally, we demonstrated the synthesis of the Dx:Fe_3_O_4_ composite in several steps using the chemical technique of FTIR and UV–Vis spectroscopy. The FTIR spectrum revealed specific band shifts from 528 cm⁻^1^ to 564 cm⁻^1^, suggesting coordination between Fe ions and hydroxyl groups in dextran. The absorption band at 1151 cm^−1^ indicate the presence of Fe-O bond. The absorption spectrum of the Dx:Fe_3_O_4_ composite shows an absorption maximum at 227 nm that corresponds to a shift of the 216 nm band seen in pure dextran. The absorption spectra also revealed maxima at 264 nm and 340 nm that could be addressed to electronic transitions involving Fe^3+^ ions and the presence of specific surface states or defects in the nanoparticles or to charge transfer between functional groups of dextran and Fe_3_O_4_ NPs. The photophysical processes of organic molecules interacting with light are illustrated with a Jablonski diagram, which is used to describe the light absorption and emission processes. Also, we obtained ground state bleaching bands (GBS), absorption in excited state (ESA), and, at longer wavelengths, stimulated emission (SE) with the transient absorption. Photophysical parameters such as singlet and triplet values of the lifetimes, together with the fluorescence and triplet quantum yields were determined.

## Figures and Tables

**Table 1:** Fluorescence lifetimes of dextran and Dx:Fe_3_O_4_ composite.

Sample	τ_1_ (ns)	τ_2_ (ns)	a_1_ (%)	a_2_ (%)	Φ (%)
Dx	2.18	9.24	27.41	72.59	0.07
Dx:Fe_3_O_4_	1.18	4.44	48.60	51.40	0.24

## Data Availability

Data will be made available on request.
